# Social embeddedness of pig value chains in Myanmar and its implications for food and nutrition security

**DOI:** 10.1007/s12571-022-01278-9

**Published:** 2022-03-24

**Authors:** Ayako Ebata

**Affiliations:** Institute of Development Studies, Brighton, UK

**Keywords:** Value chains, Asia, Myanmar (Burma), Food safety, Food security, Food systems, Transformation

## Abstract

Agricultural intermediaries – traders and middlemen/women – play a critical role for food security in low- and middle-income countries (LMICs). Yet, their role in improving or undermining food safety, an indicator for food quality, is not well understood. As middle-class citizens increasingly demand high-quality perishable and nutritious produce, food safety has become an important issue in LMICs. The existing literature offers limited insights as to whether and, if so, how intermediaries manage information regarding food safety in LMICs. This article fills this gap based on an in-depth qualitative study on pig value chains in Myanmar. We document that intermediaries helped reduce transaction costs of trade by linking farmers to buyers based on an intricate socio-economic relationship. While we find no evidence of intermediaries actively concealing facts about invisible (i.e. microbiological or chemical) nature of pig products, they facilitated selling sick animals. On the other hand, intermediaries withheld information about potential buyers and sellers in order to maintain their role along the value chains. In order to improve food safety in LMICs, policies need to reduce transaction costs of trade as well as access to public health expertise.

## Introduction

Agricultural intermediaries – traders and middlemen/women – play a critical role in food security and overall economic development of the agricultural sector across low- and middle-income countries (LMICs) (Reardon, 2015). Large-scale investment in wholesale markets, processing and storage technologies and rural infrastructure has improved productivity at the processing level, and increased the supply of high-quality agri-food commodities across many LMICs, particularly in Asia (Reardon & Timmer, 2014; Reardon et al., 2012). Such transformation has been critical in supplying perishable and nutritious products such as fruits, vegetables and animal products whose demand has increased due to rapid urbanization and economic growth (Belton et al., 2020; Huynh et al., 2007; Philipsson et al., 2011). Particularly the push from better-off urban consumers has incentivized investment in the middle segments of value chains to improve efficiency of agricultural commodity trade in LMICs (Pingali, 2007).

While increased consumption of fresh and nutritious foods is critical to nutrition security, the increased demand for perishable foods – particularly animal-sourced food (ASF hereafter) – has been linked to increased incidence of gastrointestinal illness that hinders cognitive and physical development (Jones et al., 2013) and the emergence of infectious diseases such as avian influenza (Gilbert et al., 2017). Myanmar, where this study was conducted, demonstrates a high risk of animal-borne diseases because of rapidly increasing demand for ASF and poverty among livestock producers (ILRI, 2012). While food safety issues in Myanmar remain less political than neighboring countries such as Vietnam (Nguyen-Viet et al., 2017) or China (Cicia et al., 2016), Myanmar’s livestock value chains are facing the challenge of increasing meat supply in an efficient and safe manner (Myint, 2019).

In many LMICs where livestock demand is increasing, traceability is often lacking and unsafe food can be easily traded (Chen, 2008; Indrawan et al., 2018; Tornimbene et al., 2014) where intermediaries play a role in (mis)communicating product quality (Minten et al., 2017). This can lead to the consumption of food contaminated with harmful microorganisms and mycotoxins, affecting the health of the public (Grace et al., 2015; Salmon et al., 2018) and food and nutrition security. While agricultural intermediaries are deemed key to improving food safety (Alarcon et al., 2016; Fournie et al., 2013), how they manage food safety is so far undocumented.

Against this backdrop, this article examines the roles of agricultural intermediaries in supplying safe ASF in LMICs based on a case study from Myanmar. Specifically, we focus on the socio-economic relationships that intermediaries develop and utilize in order to facilitate the trade of ASFs, and what consequences this has on food safety. In LMICs, information asymmetry and missing input markets (e.g. for credit) make economic transactions costly. Intermediaries use social networks to reduce transaction of commodity trade by sharing market information, providing credit and ensuring the quality of products (Fafchamps & Minten, 2002). While existing research commends how traders’ social networks contribute to economic development and food supply (Fafchamps & Minten, 1999, 2002), it lacks evidence regarding how livestock traders – and value chain more broadly – can help improve food safety (Hatab et al., 2021). The empirical evidence in this paper indicates that while intermediaries do not actively conceal information about invisible characteristics of pig products, the intricate socio-economic relationships between intermediaries and other value chain actors incentivize them to facilitate selling diseased pigs.

The rest of the article is organized as follows. In Sect. 2, I outline the theoretical framework that helps analyze the role of agricultural intermediaries in supplying healthy pigs in Myanmar and the data collection and analysis methods. In Sect. 3, the empirical results are presented to discuss how intermediaries reduced the transaction costs of pig trade as well as veterinary healthcare and input supply that farmers needed, how the intricate socio-economic relationships between intermediaries and farmers influenced the economic wellbeing of farmers as well as the quality of pig products exchanged. In Sect. 4, I discuss policy implications of the findings before concluding in Sect. 5.

## Materials and methods

### Theoretical backgrounds

Economics literature argues that intermediaries emerge along agri-food value chains due to high transaction costs of commodity trade (Williamson, 1981). Transaction costs refer to the cost of acquiring the information needed to exchange products, negotiating the terms of trade, as well as the monitoring and enforcement of the agreed terms (Hobbs, 1997). These costs tend to be high in LMICs because the production segment of value chains tends to be fragmented: in other words, a large number of farmers sell a commodity without being organized through, for instance, cooperatives (Lapar et al., 2010). This, in turn, increases the cost of searching for a buyer on the farmer’s side and a seller on the buyer’s side (Gabre-Madhin, 2001b). As a result, intermediaries, who specialize in commodity trade, improve the efficiency of trade by connecting willing sellers and buyers across a wide geographical area (Fafchamps et al., 2005; Gabre-Madhin, 2001a, 2001b; Kopp & Brümmer, 2017; Minten et al., 2011; Reardon et al., 2014).

When the quality of exchanged goods improves because, for instance, consumers demand higher quality food than before, transaction costs for value chain actors increase (Humphrey & Schmitz, 2002). Food is safe when it is free from harmful chemicals and microbes (Abdulai & Kuhlgatz, 2012). In this context, improved quality (i.e. safety) of food is a *credence characteristic* that is not easily observable by buyers at different stages of value chains. This creates *information asymmetry* (Akerlof, 1970) between sellers, who have an overview of the credence characteristics, and buyers, who lack such information. Due to the lack of appropriate monitoring and enforcement in Hobbs' (1997) terminology, consumers in LMICs are often unaware of biological and chemical quality of agricultural produce (Ortega & Tschirley, 2017). In turn, value chain actors can exploit the information asymmetry for economic gains (Minten et al., 2016; Xiu & Klein, 2010) as consumers’ assessment of product quality is limited to visible, rather than credence, characteristics in LMICs (Reardon et al., 2012).

In this article, I argue that reducing transaction costs and information asymmetry regarding product characteristics is only a partial explanation to why agricultural intermediaries exist. Instead, they play a wider set of roles as *socially embedded* actors. In other words, agricultural intermediaries interact with farmers and buyers not only for economic activities but also in socio-cultural settings (Abebe et al., 2016). While social ties can reduce transaction costs and help individuals cope with economic risks (De Weerdt & Dercon, 2006; Fafchamps & Minten, 2002), they can make the powerless vulnerable to the risk of exploitation by the powerful (Granovetter, 1985; Sayer, 2001).

Examining the social embeddedness of agri-food value chains is critical for three reasons. First, the social embeddedness of livestock markets encourages intermediaries to facilitate the trade of sick animals as this is critical for them to maintain socio-economic ties to actors upstream and downstream. This is an example of how social networks can affect people and businesses negatively (Portes, 2014). For instance, Levine et al. (2014) document that ethnic ties inflate stock prices because individuals who trade place excess trust in others when they come from the same ethic community. Likewise, de Vaan et al. (2019) show that social networks can inhibit entrepreneurs and prevent new industries from emerging because such innovation is against the socially accepted “norms”. In this paper, I demonstrate that the socio-cultural ties that enable economic transactions along Myanmar’s pig value chain encourages intermediaries to trade sick animals.

Secondly, the socially embedded relationship between farmers and intermediaries can lead to exploiting farmers, who are relatively powerless compared to intermediaries, and thus diminishing their financial ability to invest in production technologies that can help produce safe animal products. Economic sociologists have long argued that social relationships do not always imply a harmonious and mutually beneficial relationship (Hinrichs, 2000). While social networks can benefit individual actors, social relationships may exist between parties with imbalances of power and in turn, can make the party with less power and resources vulnerable to exploitation by the other (Bowen, 2011). Similarly, trust may be derived from, for example, a lack of alternatives for the powerless where they have no choice but to cooperate with the powerful (Sayer, 2001). These sociological insights caution against a naïve understanding of social networks as an amicable mechanism that helps reduce transaction costs and information asymmetry to the benefit for all parties.

Third, understanding the range of services that intermediaries provide farmers allows us to identify the structural constraints in producing safe animals for human consumption in LMICs. While much of the existing policy advise focuses on improving stakeholder knowledge and incentivizing good practices through product certification, these mechanisms have yielded limited results (Grace, 2015; Grace et al., 2015; Nguyen-Viet et al., 2017). This study contributes to this debate by highlighting the social mechanisms that counteract structural constraints, their advantages and disadvantages, and how they can lead to improved food safety in LMICs.

Based on an in-depth qualitative data from pig value chains in Myanmar, I address the following objectives in this paper: 1) to evaluate the roles played by pig intermediaries and the specific socio-economic contexts that encouraged their emergence; 2) to critically examine whether the social relationships between farmers and intermediaries embody power imbalances and potentials – or examples – of exploitation of the powerless by the powerful; and 3) to analyze how the socio-economic relationships between farmers and intermediaries influences food safety. The analysis focuses on the incidence of trading sick pigs as a proxy for unsafe food as no microbiological or chemical evidence is available to detect their actual prevalence in traded meat.

### Methods of data collection and analysis

The data were collected through qualitative methods – rather than quantitative – as the aim is to understand intermediaries’ practices and rationales behind them in depth. We collected qualitative data in three townships in Myanmar’s Yangon Region, which has an active pig industry (LBVD, 2014). Hlegu, a peri-urban township about 40 km from Yangon, has relatively large-scale pig farms with 70 or more pigs including some of the largest pig farms in Myanmar with thousands of pigs reared at a time. Most pig farms in Hlegu are commercial farms, raising industrial breeds with commercial inputs. Taikkyi, a rural township located about 70 km from Yangon, has predominantly medium-scale commercial farms with between 30 and 70 pigs. South Dagon, 30 km from Yangon in an urban area, has small-scale farmers who keep less than 30 local breed pigs at a time. In South Dagon, there is a large-scale slaughterhouse where several hundred pigs are slaughtered every day while slaughterers in Hlegu and Taikkyi slaughter up to 10 pigs per day in various locations across the townships.

Between June 2016 and May 2017, we conducted individual semi-structured interviews, focus group discussions (FGDs), and participant observation on farms. Our data collection mostly focused on farms as our research was conducted as part of a large interdisciplinary project on pig-borne zoonotic diseases in Myanmar. We selected a total of 28 farmers who represented a typical farming style for their township and who considered pig farming to be an important income source. We sought diversity in terms of a farmers’ gender and farm size whenever possible. We then applied a snowballing sampling approach and identified traders and middlemen, slaughterers, retailers and consumers who interact with the selected farmers.

In total, we interviewed 28 farmers, 3 traders, 12 slaughterers, 12 pork vendors and 12 pork consumers. In addition, we conducted a total of 12, 6 and 6 FGDs with farmers, consumers and community members who are actively participating in product intermediation. We continued data collection until we reached information saturation (Guest et al., 2006) where additional interviews did not yield any new information. Our data collection addressed the understandings of pig diseases by individuals, measures taken to control pig diseases, and how information regarding pig health and pork quality is communicated along the value chain. In addition, two researchers (including the author) spent two days at each farm to conduct participant observation between December 2016 and February 2017. Through observation and informal discussion, we gathered information regarding marketing practices by farmers and observed how pig trading is conducted in study sites. To gain a national-level overview, we also conducted additional interviews with two policy makers from the Livestock Breeding and Veterinary Department (LBVD) of the Ministry of Agriculture, Livestock and Irrigation (MOALI) in November 2019.

The analysis is based on transcribed interviews and FGDs as well as the fieldnotes recorded during observation. We systematically coded the data according to relevant themes including: stakeholder understandings of food safety; the patterns of pig trade; farmers’ and slaughterers’ rationale to employ traders and middlemen; socio-cultural relationships between intermediaries and other value chain actors; and the wider contexts of animal health management and veterinary healthcare provision. All analysis was conducted in Nvivo (Version 12), a qualitative data analysis software.

## Results

### Facilitating trade: linking sellers and buyers

**Fig. 1:**
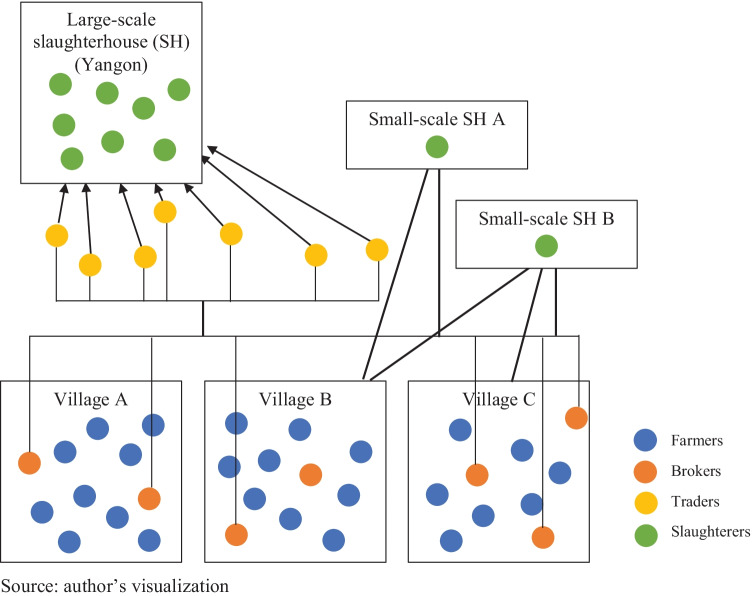
Typical pig trading channel in the Yangon region

Pig value chains examined demonstrated the challenges that led to high transaction costs for farmers and buyers. Across the three townships, there was no product differentiation through, for example, branding pork and no farmer cooperative assisted farmers in marketing their pigs. In this context, farmers relied on *brokers*, who lived nearby, to trade pigs (Fig. 1). Farmers kept phone numbers of several brokers whom they called to get a sense of pig prices and buyers. When farmers decided to sell their pigs, they contacted one of the brokers in their village. Because brokers communicated with multiple buyers, farmers could compare prices and find someone who was willing to purchase pigs in a timely manner. Then, brokers contacted *traders* and communicated the location of the farm, number of pigs and the characteristics of pigs. In other words, traders and brokers – pig buyers hereafter – facilitated pig exchange by linking sellers and buyers who would have otherwise not interacted.

Intermediaries emerged because all slaughterers were mandated to process a certain number of pigs every night.^1^ Therefore, they paid traders to purchase the required numbers of pigs every day as demonstrated in the below quote:

*“We cannot run our business without traders. They gather pigs in this [agreed] location. If we tell traders from War Khae Ma to gather so and so many pigs, they will go and buy from other places and will gather all those pigs in War Khae Ma. We just have to go there and buy the pigs from them.” (Slaughterer 811, large-scale).*

Large-scale slaughterers, who supplied pork to urban markets as well as supermarkets in Yangon, had to slaughter 30 or more pigs a day while small-scale slaughterers in townships outside Yangon only needed between one and 10 pigs. As a result, small-scale slaughterers purchased pigs directly from villagers or through local brokers. Large-scale slaughterers requested traders in nearby Yangon and Bago regions and from remote Rakkhine and Ayeyarwady regions to collect a certain number of pigs. Traders then transported purchased pigs to an agreed location. Slaughterers considered local traders crucial in securing the required number of pigs every day.

Traders’ social networks with farmers and brokers were crucial to stabilize the supply of pigs to slaughterers:

*“[Whether I can buy enough pigs or not] depends on business and social relationships between farmers and us. They come to us if they want to buy weaned piglets.*^2^* So, we can guess when they might sell the pigs. [When we think it might be the right time for them to sell pigs] we call them and ask if they want to sell the pigs soon.” (Trader 1).*

In addition, traders relied on brokers in local areas. As depicted in Fig. 1, the use of traders was more common along value chains that catered to large-scale slaughterers in Yangon who supply pork to urban consumers than through local value chains. These traders needed to supply 40 to 50 pigs a day to large-scale urban slaughterers. As traders had to travel to multiple locations to acquire the required number of pigs, they relied on brokers to secure pigs for them to purchase and transport prior to their visit.

For urban value chains, brokers played an important role in not only securing pigs but also assessing the conditions of pigs:

*“We hire middlemen [brokers] to check pigs’ conditions whether their flesh is suitable or healthy. And that person sets a good price. Otherwise, we cannot trade efficiently by ourselves as the farms are quite far away and we need a lot of time to visit them.” (Trader 2).*

When brokers went to farms prior to a trader’s visit, they checked the breed and flesh of pigs and agreed on the price and timing of trader’s visit. Due to the preference by urban consumers, they sought out pork without fat and bones:

*“It is important for us [to buy improved breeds] as we sell fat and meat separately when we sell pork. It has different prices between fat and meat. If the meat is 10,000 kyats*^3^* for one viss,*^4^* we can get only 2000 kyats for one viss of fat. So, we differentiate beautiful pigs by the amount of meat and fat in the body.” (Trader 1).*

Our findings indicate that the transaction costs of finding pig sellers on the part of urban slaughterers resulted in layers of intermediaries who link rural farmers to urban slaughterers by collecting a large number of pigs from many farmers. In addition to facilitating trade, intermediaries responded to the information asymmetry regarding product quality (e.g. pig breed, fat to meat ratio) and price between slaughterers and farmers.

### Food safety and product quality judgement

Many governments in LMICs, including Myanmar, face difficulty in providing services that monitor the quality of animal products due to limited capacity and infrastructure (Haggblade et al., 2014; Häsler et al., 2017). Indeed, the hygiene of farms and slaughtering premises, pathogen prevalence in meat, and antimicrobial use before slaughter were not adequately monitored by inspection agencies throughout the value chains. No participant mentioned that meat samples were collected from markets to detect biological contamination. Regulations exist regarding ante- and post-mortem inspection of pigs and the government has recommendations regarding good farming practices. However, the implementation and enforcement of these measures was rare.

In a context where food safety surveillance is limited, thereby information about credence nature of food safety is unavailable to consumers and value chain actors, food safety and local people’s judgement on product quality are not entirely aligned. Food is unsafe when it is contaminated with harmful organisms or chemicals (WHO, 2019), most of which are invisible. As a result, consumers had limited means to judge food safety. To judge the *quality* of pork, consumers turned to observable characteristics such as color, taste and texture:

*Interviewer: How do you differentiate good meat from bad meat?*

*Consumer A: Good meat is red.*

*Consumer B: We don’t buy meat if it is turning white.*

*Interviewer: What else?*

*Consumer A: I also check whether the meat is hard or soft. Some [bad] meat is soft.*

*Consumer C: There is no taste if we cook bad meat. And the color is also a little bit brown.*

*(Consumer FGD 401)*

Even though it was common that consumers judged meat quality based on visible observations, they also recognized the limitation of their judgement.

*Interviewer: Have you ever heard about people selling pork from dead or unhealthy pigs?*

*Consumer B: Yes, I heard about that… I think they sell [such meat]. We wouldn’t know even if they [vendors] sell pork from unhealthy or dead pigs… We check the meat and, if the color is not good, we don’t buy.*

*Consumer A: I think so too [vendors sell such meat]. That’s why we hardly ever go to that market. I don’t know exactly which shops sell such meat. We can’t see it.*

*(Consumer FGD 401)*

Similarly, pig buyers relied on visible characteristics of pigs when purchasing pigs from farmers. They slapped pigs to see whether pigs moved quickly or not: if not, they might be ill.

Despite the limitations of judging quality based on visible characteristics, actors throughout the value chains suggested that consumer judgement significantly influenced their behavior.

*“… we do not accept ill pigs. I have told them [traders] in advance that I would not buy ill pigs… Ill pigs have bad odor. We can’t sell that kind of meat because it leads to bad reputation among my customers.” (Slaughterer 801, small-scale).*

*“We don’t buy sick pigs because we are a big (pork) seller in Hlegu and if we buy bad pigs and sell the meat, we will get bad reputation. We are not brokers. These customers buy pork from us repeatedly…Also, we live in the neighborhood. We don’t want to get bad reputation from the customers. So, we don’t buy sick pigs.” (Vendor 605).*

Their main concern was consumer mistrust toward product quality as this can lead to food safety scandals and reputational damage for the whole industry (Xiu & Klein, 2010). In Myanmar, panic amongst consumers during disease outbreaks led to dramatic decrease of pig prices, affecting businesses along the value chains:

*“When there was Blue Ear [common name for porcine reproductive and respiratory syndrome (PRRS), not zoonotic], no one ate pork for one or two months and pork price dropped drastically. Normally, a pig would be valued at 2,800MMK*^5^*/viss but at that time, it dropped to 500 or 600MMK/viss.” (Slaughterer 812, large-scale).*

The above evidence suggests that consumers and value chain actors had some means to judge product quality and avoided purchasing visibly sick or dead pigs. However, consumer judgement and supplier knowledge on pork safety was limited to visible characteristics of meat and pigs as information regarding invisible characteristics was unavailable. In other words, intermediaries were equally unaware of credence characteristics of pig health and therefore did not deliberately withhold such information from consumers or other value chain actors.

### Balancing trade facilitation and food safety

If value chain actors were careful not to trade sick pigs, why were they still traded? Understanding the rationale highlights the critical role played by intermediaries. First, and perhaps most importantly, value chain actors accepted that trading sick pigs was the norm and unavoidable:

*Interviewer: So, within one year, from last October to this October, have you slaughtered any sick pig in your slaughterhouse?*

*Slaughterer: Yes. As we both know, our country is poor and…they [pigs] get sick frequently. So, I always tell the pig sellers from Phaung Gyi and Mingone – I know them very well – ‘Tell me once your pigs stop eating. I won’t buy them if it’s too late and they are very sick. Customers know when we sell meat from diseased pigs. Even when they buy it, it’s not a good thing because the disease will spread widely.’ I have to tell them honestly because things like that can happen and we don’t want to lie. We are poor so we can’t waste anything.*

*Interviewer: So, according to what you said, you kill pigs when they stop eating?*

*Slaughterer: Yes, if a pig doesn’t eat today, it is killed tomorrow. And there won’t be any problem.*

*Interviewer: So, how about the pigs that are already sick? You don’t slaughter them if it’s too late and they are already sick?*

*Slaughterer: No, sick pigs cannot be slaughtered. We would get into trouble when the vet investigates. The vet comes here and investigates.*

*(Slaughterer 806, small-scale)*

This conversation indicates that the slaughterer acknowledged that sickness among pigs is common. He defined that the severity of illness increased with the number of days since pigs had stopped eating. As a result, he advised his “pig sellers” to inform him when pigs stopped eating so that he could slaughter them before they became “very sick”. He justified this act by saying that because Myanmar is poor, nothing – including sick pigs – can be wasted. This insight from the slaughterer explains why farmers would try to sell pigs that lose appetite as soon as possible.

Secondly, even though most consumers avoided what they perceived to be pork from sick or dead pigs, some – poorer – consumers purchased such pork. Loss of appetite, seasonal fever and physical injuries were considered as “minor illness” by slaughterers and traders:

*“If the pig is seriously sick, we don’t slaughter them, but we slaughter the pigs that are only not eating.” (Slaughterer 807, small-scale)*

As a result, they slaughtered pigs that suffered from minor illness and sold their meat at a cheaper price:

*Interviewer: What time of the year do you find sick pigs?*

*Slaughterer: When the season changes, from summer to rainy season. I think it was just fever because of heat. They [these pigs] started to shiver and died suddenly.*

*Interviewer: How did you handle those pigs? Did you mix meat from those pigs together [with meat from other – healthy – pigs] or did you separate it?*

*Slaughterer: We have to separate it and give it to people who ask for meat from dead pigs… We have to sell such meat at a lower price.”*

*(Slaughterer 812, large-scale)*

Because trading sick pigs was considered customary, traders and brokers facilitated the trade. The question was no longer “is this pig sick?” but rather “*how sick* is this pig?”. Based on their perceived degree of illness, traders negotiated prices on behalf of slaughterers:

*“Buyers [Traders] decrease the price when they hear about pig diseases around here. Even if individual pigs are healthy, they only buy at a lower price to get profit.” (Farmer FGD 211).*

In doing so, traders ensured that slaughterers only pay prices that are considered fair based on their local knowledge:

*“If I go to villages to buy pigs myself, I could lose my money… so, I order from the trader according to the current price. Then he buys pigs from the region as he is familiar with the area. It’s always better to work like that. If I go myself, everyone will lie.” (Slaughterer 812, large-scale).*

As the above quote indicates, slaughterers valued traders’ social network that helped ensure that the agreed prices were fair in the local context.

In addition, brokers negotiated prices for farmers. Observations across the three townships revealed an intricate socio-economic relationship between farmers and brokers who played multiple roles for farmers. Because access to certified veterinarians from the government was limited, most farmers relied on other farmers – locally referred to as “*wa saya* (pig masters)” – and/or Community Animal Health Workers (CAHWs) to treat illness in pigs. These pig masters who were providing veterinary healthcare often played a role as brokers as well. They actively supported farmers in negotiating the price of pigs offered by traders.

To illustrate the roles of a broker, let us describe an incident where a sow^6^ was sold after a complicated delivery. A farmer – let us call him Ko Kyaw – acted as a pig master and broker in the neighborhood. One day, he was asked to assist with labor by a farmer whose sow conceived with Ko Kyaw’s boar.^7^ He went to assist the delivery as it was customary for boar owners to take care of sows impregnated by their boars. He was to provide other services to the new-born piglets including vaccination, cutting teeth and tails and regular monitoring of piglets’ health. It was a stillbirth and the sow did not deliver the placenta. Ko Kyaw warned the owner that there might be some unborn piglets left in the womb and recommended him to sell the sow immediately. The owner decided to follow the advice: otherwise, he could not retrieve any revenue. Quickly, Ko Kyaw called a trader to visit the farm. Minutes after the phone call, a group of traders arrived at the farm to negotiate the price. The traders asked why the owner wanted to sell the sow and were concerned that the sow was so sick that it would die on the way to the slaughterhouse: this would result in financial loss for the traders. Ko Kyaw explained that he has manually extracted all stillborn piglets from the womb and has given appropriate treatment to avoid death. The traders agreed to buy the sow on the condition that the sow survives until the next day. After the traders left, Ko Kyaw injected more medicine, saying that this would help the sow live until the next day, when the traders would return to the farm.

Ko Kyaw’s negotiation was effective. On this occasion, the sow owner received 310000MMK for the sow. In this locality, a typical selling price for a sow was between 180000MMK and 700000MMK. The lowest was recorded during the disease season, in June and July, when traders were known to offer lower-than-usual prices. For local breeds, a healthy sow was sold by another farmer at 350000MMK. In this context, the price that the sow owner received was slightly lower than usual.

For assisting in the delivery, Ko Kyaw only requested the sow owner to pay what he could afford. Ko Kyaw confessed that his treatment was perhaps inadequate. As a token of appreciation, the owner paid 10000MMK, which Ko Kyaw shared with another person who helped him in assisting with the delivery. In this locality, farmers typically paid 15,000MMK for assistance with deliveries. Given that the sow owner lost both piglets and sow, Ko Kyaw perceived that this payment was adequate. Across the study sites, we observed several of these multi-role brokers. They considered that providing healthcare to pigs, renting a boar and facilitating pig trade was an important means to maintain good relationships with farmers. Providing “a package” of services, pig farmers tended to turn to particular individuals when they required assistance.

This socio-economic relationship between brokers and farmers explains why this layer of intermediation continued to exist. Several farmers complained about the intermediation fees charged by brokers, because brokers sometimes refused to link farmers and traders:

*“Traders don’t come here often because brokers don’t give [our contact] information to them.” (Community FGD 906)*

*“We usually sell through brokers because it is convenient…Sometimes, traders came [to the farms] directly. But if you use a broker, both buyer and seller have to pay them. If we were connected with traders directly, we wouldn’t need to pay.” (Community FGD 904).*

Another strategy used by brokers was to create a price difference between farmers and traders or slaughterers, or to charge intermediation fees per pig traded:

*“Brokers bought [pigs] for 47,000MMK per viss from farmers but sold them for 47,500MMK per viss…Others ask 3,000MMK per pig. So, they got 30,000MMK for 10 pigs.” (Licensee 804).*

However, farmers still used brokers, because they provided other services that were vital to their pig farming enterprises.

The evidence presented above highlights two key points. First, value chain actors ran their businesses on the notion that sick pigs can be sold if the condition is not “severe”, thereby encouraging them to trade pigs that are becoming ill quickly. This was their strategy to avoid long-term reputational damage through trading severely sick pigs while maintaining their business operation and thereby earning income. Second, traders and brokers capitalized on information asymmetry and earned living. Traders were extremely mobile, and thereby more familiar with the distribution of farms and disease dynamics than slaughterers or farmers. As a result, they earned a margin by facilitating trade. Brokers were more aware of the details of pig health, including the history of care and health, than traders and slaughterers. In addition, they deliberately withheld information about traders and slaughterers from farmers. Based on these two types of information asymmetry, brokers retained a share of profit along the value chain.

## Discussion and policy implications

This study provides a unique insight to the way in which food safety and food supply are managed by intermediaries in LMICs. As in many LMICs (FAO, 2019; IFAD, 2013), the majority of fresh agricultural commodities in Myanmar is produced, processed and distributed by small-scale and resource-constrained actors. These actors lack the capacity and incentives to enforce quality standards, and the government or any external actors are currently unable to provide services that would ensure pig health and safety of pork. Moreover, extension services to livestock farmers are limited (Haggblade et al., 2014). As a result, veterinary healthcare access including diagnostic tools among small-scale farmers remains lacking.

Pig intermediaries operate in a context where the pig industry is facing a tension between increasing the supply and safety of pork and ASF, which translated to a trade-off between two competing requirements for traders and brokers. The first is to avoid “the risk of commitment failure” (Gabre-Madhin, 2001a, p.36) where intermediaries are required to supply a given number of pigs in a given time frame. While none of the traders had contracts with slaughterers that detailed the nature of trade, fulfilling this task for the urban market was important in maintaining the business relationship with slaughterers based on trust. The second requirement was to avoid reputational damage caused by purchasing pigs that were “too ill”. Here, the emphasis on the degree of illness allows for strategic ambiguity where intermediaries could balance ensuring pig supply to slaughterers and helping farmers sell their pigs. As quality monitoring was virtually nonexistent throughout the value chains, consumers had no means to assess pork safety in a microbiological or chemical sense. However, they judged product quality based on observable characteristics so traders had to purchase sick pigs that would not signal severe illness in the eyes of consumers.

Intermediaries managed the trade-off between pork safety and efficient pork supply pragmatically. Although value chain actors were aware of disease patterns to a limited extent, the details of pig illness and its implications on public health – for instance what pathogens cause what illness, and what this means to people – were unknown to most, if not all, actors. In this context, traders and brokers focused on managing the risk of undersupply. As a result, sick pigs were sold before illness became severe. Trading sick pigs was, in their mind, beneficial for all value chain actors: farmers were able to sell sick pigs, slaughterers processed the meat as mandated, and poor consumers could afford pork that was otherwise out of reach. In Myanmar, people cook meat at a high temperature to make popular curry dishes. This cooking method was perceived to make food safe by many consumers, even when meat from pigs considered to be “too sick” was sold to poorer consumers.

Intermediaries emerged because of the structural constraints in providing timely veterinary healthcare, fragmented markets, locally specific disease dynamics, and the transaction costs for farmers to obtain all the necessary services to raise pigs in resource-poor settings. The need to overcome these challenges motivated intermediaries and farmers to form an intricate socio-economic relationship that was perceived to benefit both. Because farmers lacked access to potential buyers and qualified veterinarians, they relied on brokers who offered a “package” of services that oversaw pigs from birth to sales. This reduced transaction costs for farmers as they could rely on one individual to take care of pigs at different stages of growth. As a result, farmers kept paying the intermediation fee to maintain a good relationship with brokers who treated their pigs. Brokers actively served farmers by facilitating pig sales because farmers preferred to turn to those individuals when their pigs fell ill. When brokers, who were also pig masters, got paid by farmers for providing veterinary health care, they benefited from the information asymmetry regarding the history of health and care of a particular pig between farmers and traders, and obtained clients who would provide them with an income opportunity.

While brokers certainly helped reduce transaction costs for farmers, they retained a level of information asymmetry to ensure additional income generating opportunities for themselves. This behavior is well documented in economic sociology: individuals who share economic interests often interact in a particular social context, which can make the party with less information access and resources vulnerable to exploitation by the other (Granovetter, 1985; Hinrichs, 2000). Our study context demonstrated information asymmetry regarding pig supply and demand, observable pig quality, and prices regarded as fair by various actors in a particular geographical area along the value chains. Intermediaries could profit from such information asymmetry to gain profit from facilitating pig sales even though the commission fee was not unreasonable.

Without microbiological evidence, we are unable to assess how these behaviors by intermediaries affect food safety. However, our evidence highlights the importance of reducing transaction costs of trade through investment in information and transportation infrastructure (Abdulai & Kuhlgatz, 2012) as well as to strengthen veterinary health services and capacity in animal production that lead to safer ASF supply. Such investment will likely make the role of intermediaries less important for farmers’ and slaughterers’ business operations. In turn, this can increase the share of profit margins along the value chains retrieved by pig farmers. This will increase their income, and thus financial ability, to invest in pig health management and thereby improving livelihoods as well as food safety. Likewise, slaughterers may become incentivized to improve slaughtering conditions if they no longer need to pay fee to intermediaries. Simultaneously, interventions are needed to inform consumers as well as value chain stakeholders about the credence characteristics of pork exchanged throughout the value chain. This includes, but is not limited to, signaling pork quality through certification or branding and microbiological testing at key nodes such as slaughterhouses.

## Conclusions

Our study analyzed pig trade in Myanmar, a country that was going through rapid economic growth, urbanization and lifestyle change until the 2021 coup. In a context where food safety monitoring is limited yet the public increasingly demands improved food safety, value chain actors operated on the understanding that trading sick pigs was unavoidable. To facilitate the efficient trade of “not-so-sick” pigs, an intricate socio-economic relationship emerged between brokers and farmers, and to a lesser extent, traders and slaughterers. The socio-economic arrangement helped to not only reduce transaction costs of the pig trade, but also those of veterinary services required by farmers.

While the actors in the intermediate segments certainly facilitated pig trade, our evidence suggests that the information asymmetry – regarding pig supply and demand, observable pig quality, and prices – generated an opportunity for intermediaries to gain extra income. Because farmers faced high transaction costs of pig production and commercialization, they relied on brokers who were reluctant to directly link farmers and traders in order to retrieve income. Another concern of the current marketing mechanism is the trading of sick pigs. Value chain actors considered this a beneficial outcome for all value chain actors and consumers, because pigs often fell ill for various – albeit not well understood – reasons. As there is limited research on health burden from consuming sick animals, the implications of such practices on food safety as well as nutrition and food security need to be carefully evaluated in future research.

We argue that LMICs need explicit strategies that target improvement in food safety as well as food and nutrition security simultaneously, and ways to incentivize measures that could be taken by intermediaries. The current policy and academic debates emphasize food safety concerns for export markets while neglecting the importance of domestic agri-food value chains that provide ample and safe foods to their dynamic populations. At a time when consumers are increasingly conscious of food safety issues (Ortega & Tschirley, 2017), food policies should target improving food safety of ASF consumed in LMICs. This research shows that such effort needs to target not only awareness raising by consumers and farmers but also infrastructure development and capacity building to reduce transaction costs of trade and veterinary healthcare access and tackle information asymmetry regarding credence characteristics of ASF.

## Data Availability

As our research is based on qualitative data, specific data that corresponds to our findings can be shared upon request.

## Footnotes

1 Slaughterers obtained an annual slaughtering license from City Development Committees (CDCs) and the license specified how many pigs are to be slaughtered every day. This was to stabilize the supply and price of pork available in markets, according to CDC officials.

2 Farmers sold piglets as well as fattened pigs as this allowed them to avoid the risk of raising pigs and losing them to illness and/or when they needed access to cash. This practice was more common among small-scale farmers than large-scale farmers as they raised pigs as a saving rather than to maximize income.

3 Myanmar’s local currency. 1USD = 1,300MMK at the time of the fieldwork.

4 1 viss = 1.6 kg.

5 Myanmar kyats: 1USD = 1,300MMK at the time of the fieldwork.

6 Female pig.

7 Male pig.

## Code availability

As our research is based on qualitative data, there is no programmatic code to share. However, we have outlined the analytical framework that informed the analysis in the submitted manuscript.
